# Engaging the Community to Improve Nutrition and Physical Activity Among Houses of Worship

**DOI:** 10.5888/pcd11.130270

**Published:** 2014-03-13

**Authors:** Kiameesha R. Evans, Shawna V. Hudson

**Affiliations:** Author Affiliation: Shawna V. Hudson, Department of Family Medicine and Community Health, Rutgers Robert Wood Johnson Medical School, Rutgers, The State University of New Jersey, New Brunswick, New Jersey.

## Abstract

**Background:**

Obesity, physical inactivity, and poor nutrition have been linked to many chronic diseases. Research indicates that interventions in community-based settings such as houses of worship can build on attendees’ trust to address health issues and help them make behavioral changes.

**Community Context:**

New Brunswick, New Jersey, has low rates of physical activity and a high prevalence of obesity. An adapted community-based intervention was implemented there to improve nutrition and physical activity among people who attend houses of worship and expand and enhance the network of partners working with Rutgers Cancer Institute of New Jersey.

**Methods:**

An adapted version of Body & Soul: A Celebration of Healthy Living and Eating was created using a 3-phase model to 1) educate lay members on nutrition and physical activity, 2) provide sustainable change through the development of physical activity programming, and 3) increase access to local produce through collaborations with community partners.

**Outcome:**

Nineteen houses of worship were selected for participation in this program. Houses of worship provided a questionnaire to a convenience sample of its congregation to assess congregants’ physical activity levels and produce consumption behaviors at baseline using questions from the Health Information National Trends Survey instrument. This information was also used to inform future program activities.

**Interpretation:**

Community-based health education can be a promising approach when appropriate partnerships are identified, funding is adequate, ongoing information is extracted to inform future action, and there is an expectation from all parties of long-term engagement and capacity building.

## Background

Obesity, lack of physical activity, and poor nutrition have been linked to chronic diseases such as heart disease, stroke, diabetes, and some cancers (1). An estimated 18.4% of adolescents and 35.7% of adults in the United States are obese (2). The World Cancer Research Fund estimates that 25% to 30% of all cancers in the United States can be attributed to conditions related to diet and physical inactivity, including overweight and obesity (3). Recognizing the effect that obesity and lack of physical activity has on chronic diseases and their prevalence and outcomes, government and private organizations have developed individual- and community-level recommendations to reduce rates of morbidity and mortality (1,4). Community-level guidelines support public–private partnerships that increase access to healthy foods and provide “safe, enjoyable, and accessible environments” to increase physical activity in community settings (4). Research indicates that interventions in community-based settings such as houses of worship can build on attendees’ trust to address health issues and help them make behavioral changes (5).

## Community Context

New Brunswick is a diverse community centrally located in Middlesex County, New Jersey. Nicknamed “The Healthcare City,” New Brunswick is home to 2 academic institutions, 2 hospitals, a transit hub, and various pharmaceutical companies.

Data from the 2011 Behavioral Risk Factor Surveillance System indicated that 61.5% of New Jersey residents were overweight, 26.4% participated in no leisure-time physical activity, 31.9% ate 2 or more servings of fruit daily, and only 13.9% consumed 3 or more servings of vegetables per day (6). Cities such as New Brunswick are not immune to the national obesity epidemic, and in some cases, the data for this area indicate worse outcomes. According to 2012 data from County Health Rankings, 27% of Middlesex County residents aged 20 or older reported no leisure time physical activity, higher than the 25% New Jersey benchmark and the 21% national benchmark. With regard to obesity, 24% of county residents reported a body mass index of 30 kg/m^2^ or higher, slightly less than the New Jersey and US benchmarks of 25% (7).

The purpose of our program was 1) to establish a comprehensive program to improve nutrition and physical activity among people who attend houses of worship and 2) enhance the network of partners working with Rutgers Cancer Institute of New Jersey to provide community education. This case study highlights the successes and challenges through the development and recruitment periods of our program.

## Methods

Body & Soul: A Celebration of Healthy Living and Eating (B&S) is an evidence-based program developed by the National Cancer Institute in collaboration with the American Cancer Society, the University of North Carolina, and the University of Michigan (8–11). The goal of B&S is to improve the health of congregation members in African American churches by increasing their fruit and vegetable consumption. B&S comprises 4 “pillars” that, when engaged by a church, have been shown to improve fruit and vegetable consumption: 1) a pastor who is committed and involved, 2) church activities that promote healthful eating, 3) a church environment that promotes healthful eating, and 4) peer counseling that motivates church members to eat a healthful diet (8). Research has documented the efficacy of B&S as a dietary intervention to increase fruit and vegetable consumption (5,10,12). The Office of Community Outreach (OCO) at Rutgers Cancer Institute of New Jersey has worked with the B&S program since 2006, providing grant funds, technical assistance, or both to various churches throughout New Jersey, New York, Pennsylvania, and Delaware. On the basis of OCO’s experiences with the original B&S program, we adapted the original to meet the demographics and diversity of the greater New Brunswick community. 

The original B&S program was adapted for implementation in our diverse community in a few ways. The original B&S Program was designed for implementation in African American churches. Houses of worship in the New Brunswick area reflect the diversity of the neighborhoods in which they are located. Our goal was to create community change by implementing a program that would affect a broader community and not restrict implementation to African American churches. In addition, the original program had a strong focus on fruit and vegetable consumption. Although program staff and advisors agreed that this component should remain a focus of the program, they also felt it was important to include education on broader nutrition topics and general healthful living strategies of interest to participants. Therefore, we added education on other nutrition topics such as sodium, fat, calcium, sugar-sweetened beverages, and whole grains. We also included a stronger focus on physical activity. Experience with B&S and other research suggests that ongoing technical assistance and training would help engage lay members of the houses of worship (ambassadors) and continue their participation (5,13,14). Our adapted B&S program was designed to ensure that ambassadors would have continued interaction with consistent program staff. This program consisted of a multiphase process (phase I: monthly educational sessions; phase II: physical activity program development; and phase III: increasing access to local fruit and vegetables) where participants received hands-on education and technical assistance to deliver the program to members of their congregation and encourage them to make changes (Figure).

**Figure:**
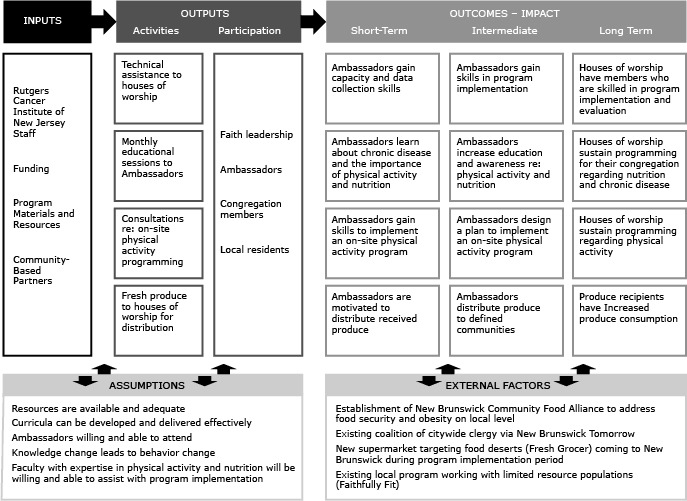
Body & Soul: A Celebration of Healthy Living and Eating logic model, New Brunswick, New Jersey, 2012.

**Table:** 

The logic model consists of a series of boxes arranged in 3 columns. The first column is labeled “Inputs,” the second column “Outputs,” and the third column “Outcomes-Impact.” An arrow points from the “Inputs” column to the “Outputs” column, and another arrow points from “Outputs” column to the “Outcomes-Impact column.” The “Inputs” column consists of 1 box and contains a list of 4 items: 1) Rutgers Cancer Institute of New Jersey staff, 2) Funding, 3) Program Materials and Resources, and 4) Community-Based Partners.
The “Outputs” column contains 2 subcolumns of boxes. The first subcolumn is labeled “Activities” and contains 4 boxes with the following text: Box 1) “Technical assistance to houses of worship,” Box 2) “Monthly educational sessions for ambassadors,” Box 3) “Consultations regarding on-site physical activity programming,” and Box 4) “Fresh produce to houses of worship for distribution.” The second subcolumn is labeled “Participation” and contains 1 box with a list of 4 items: 1) Faith leadership, 2) Ambassadors, 3) Congregation members, and 4) Local residents.
The “Outcomes-Impact” column has 3 subcolumns with 4 boxes in each. The first subcolumn is labeled “Short-Term,” the second “Intermediate,” and the third “Long-Term.” The boxes in the “Short-Term” subcolumn contain the following text: Box 1) “Ambassadors gain capacity and data collection skills,” Box 2) “Ambassadors learn about chronic disease and the importance of physical activity and nutrition,” Box 3) “Ambassadors gain skills to implement an on-site physical activity program,” and Box 4) “Ambassadors are motivated to distribute produce.” The boxes in the “Intermediate” subcolumn contain the following text: Box 1) “Ambassadors gain skills in program implementation,” Box 2) “Ambassadors increase education and awareness regarding physical activity and nutrition,” Box 3) “Ambassadors design a plan to implement an on-site physical activity program,” and Box 4) “Ambassadors distribute produce to defined communities.” The boxes in the “Long-Term” subcolumn contain the following text: Box 1) “Houses of worship have members who are skilled in program implementation and evaluation,” Box 2) “Houses of worship sustain programming for their congregation regarding nutrition and chronic disease,” Box 3) “Houses of worship sustain programming regarding physical activity,” and Box 4) “Produce recipients have increased consumption of fruit and vegetables.”
Across the page under all the columns are 2 boxes, 1 of which spans the width of the “Inputs” and “Outputs” columns. This box is entitled “Assumptions.” The second box spans the width of the 3 “Outcomes-Impact” columns and is entitled “External Factors.” The “Assumptions” box contains the following list of 5 items: 1) Resources are available and adequate, 2) Curricula can be developed and delivered effectively, 3) Ambassadors willing and able to attend, 4) Knowledge change leads to behavior change, and 5) Faculty with expertise in physical activity and nutrition will be willing and able to assist with program implementation. Two arrows point up from the “Assumptions” box to the “Inputs” and “Outputs” columns, and 2 arrows point down from the “Inputs” and “Outputs” columns to the “Assumptions” box.
The “External Factors” box contains the following list of 4 items: 1) Establishment of New Brunswick Community Food Alliance to address food security and obesity on local level, 2) Existing coalition of citywide clergy via New Brunswick Tomorrow, 3) New supermarket targeting food deserts (Fresh Grocer) coming to New Brunswick during program implementation period, and 4) Existing local program working with limited resource populations (Faithfully Fit). Two arrows point up from this box to the “Outcomes-Impact” column, and 2 arrows point down from the “Outcomes-Impact” column to the “External Factors” box.

Our adapted program used a “train the trainer” model in which consultants with expertise in nutrition and physical activity content areas provided education to ambassadors, who were then charged with delivering health messages to their congregation using the strategies that they felt would be successful in their respective groups. Before the full launch in 2012, elements from phases I and II were piloted using 2 houses of worship that agreed to serve as a prelaunch pilot. Feedback from this pilot was used to design reporting forms, adjust the program schedule, and frame strategies for the nutrition and physical activity consultant to consider during site visits. Conversations with community partners and key informants with expertise in the content areas were used to enhance session offerings and program support in an effort to increase the likelihood of continued participation among houses of worship after the program was formally launched.

A program staff member was selected to participate in the National Cancer Institute’s Research to Reality Mentorship Program (R2R). R2R is “an online community of practice that links cancer control practitioners and researchers and provides opportunities for discussion, learning, and enhanced collaboration on moving research into practice” (15). Through monthly blog posts and cyber seminars, program staff from Rutgers Cancer Institute of New Jersey learned about national initiatives such as the B&S Community of Practice. Staff were also invited to Fox Chase Cancer Center in Philadelphia to view its implementation of B&S in Pennsylvania. Program staff were able to use this site visit to identify best practices and obtain ideas to incorporate into our adapted program.

Because this project was conducted in the context of a community philanthropic project where 1) data were collected for program evaluation purposes, not to evaluate individual outcomes, and 2) the goal was not to produce generalizable results, the project was determined not to require approval by the Rutgers University Institutional Review Board. No identifiers (ie, personal or institutional) are used in the reporting of the results of this program evaluation.

### Community engagement

Community engagement is an important piece of any community-based initiative. A committed effort was made to engage and develop relationships with local community partners, both public and private, at the onset of the program. Potential community partners were identified by using existing relationships, and an emphasis was made to connect to those who had expertise in nutrition or physical activity or who were working with houses of worship. Existing relationships were also used to establish relationships with new partners. For example, we were connected to Faithfully Fit (www.njsnap-ed.org/fit), the faith-based arm of our state’s Supplemental Nutrition Assistance Program (SNAP) educational unit, because of our strong relationship with New Brunswick Tomorrow (www.nbtomorrow.org), a citywide nonprofit that works to bring like-minded partners together to address community issues. Faithfully Fit provides education on these topics in limited-resource communities, and it had varied pretested materials available for our use at low or no cost. New Brunswick is in its catchment area, and we were able to combine our efforts with Faithfully Fit’s efforts to reach shared goals. In addition, B&S staff joined networks such as Shaping NJ (www.shapingnj.gov; a statewide public-private partnership established in the New Jersey Department of Health’s Office of Nutrition and Fitness) to learn about new organizations (national, regional, and local) that could be tapped for support and guidance, as well as best practices that could be incorporated into program planning.

### House of worship engagement

In the summer of 2011, an introductory letter was mailed to more than 200 houses of worship in the local area using a mailing list provided by an internal department at Rutgers Cancer Institute of New Jersey. Additional contacts were obtained using traditional outreach methods such as word of mouth and personal visits to houses of worship in the catchment area (5). In addition, program staff made contact with the mayor’s office to introduce the program, build awareness and support, and identify other local partners that needed to be engaged. The Office of Communications at Rutgers Cancer Institute of New Jersey developed a press release, which was shared via mail, e-mail, our institutional website, and our institutional Facebook page. Two open houses were held in December 2011 at Rutgers University to introduce the program, answer questions, meet program staff and potential applicants, and distribute the program application (Appendix A). Dates and times were staggered by time of day and day of week, to be convenient to interested participants, who included members of ministries (eg, women’s, health, youth), faith leadership, and other interested congregants).

A comprehensive application was designed to assess the capacity of houses of worship, using language and components from the original B&S program pillars. The application also allowed us to capture descriptive information (eg, institutional demographics, prior programming activities) at baseline. In addition, it provided an opportunity to introduce ambassadors to data collection activities they would be participating in throughout the program. Through this process, we were able to reflect on their previous activities and organizational climate regarding physical activity and nutrition at baseline. In addition, more than 1 congregant was invited to serve as an ambassador to increase the ability for the house of worship to remain engaged throughout the program; this strategy has been suggested in other research (14). To submit an application, a signature from a member of the house of worship’s leadership team was required so that support and awareness of the program would be built among the leadership team.

Applications were received through January 2012 and reviewed for completeness; applicants were contacted when necessary to obtain additional information. Follow-up phone calls and e-mails were sent to any house of worship that previously expressed an interest but did not submit an application. Additional outreach and phone calls from 2 community partners (Faithfully Fit and New Brunswick Tomorrow) were provided to houses of worship in the catchment area to confirm our credibility and encourage participation in the program.

## Outcome

Nineteen houses of worship in the Central New Jersey area submitted applications, and all were selected to participate in our adapted program. This number represents approximately 10% of those invited who participated. There is great racial and ethnic diversity among these houses of worship, reflecting members of African American, Hispanic/Latino, white, East Asian, South Asian, African, Middle Eastern, and Afro-Caribbean communities, and representing various faiths and denominations. Many identified multiple races and ethnicities in their congregations. Questions were included on the application to identify a house of worship’s current activities related to physical activity and nutrition. Results indicated that many had conducted health-related activities before application, including health ministries (50%), soup kitchens (17%), food pantries (50%), physical activity programs (61%), and food policies (22%). We used this information to help us develop program activities for the following phases.

### Congregation assessment

Ambassadors were provided with surveys to assess their congregation’s physical activity level and fruit and vegetable consumption before the start of the program. Questions related to physical activity behaviors and fruit and vegetable consumption were extracted from the Health Information National Trends Survey, a nationally representative biennial survey developed by the National Cancer Institute and designed to learn how Americans find, use, and understand health information (16). Questions asked about physical activity participation, intensity, and frequency; fruit and vegetable consumption (quantity); and demographic information. A mandatory orientation was held before the program start to bring ambassadors together and explain program expectations. At that time, ambassadors were trained in how to engage their congregation to obtain respondents for the survey. Questions were printed on durable cardstock to ease completion in “on the go” environments (eg, before, during, or after worship service; at weekly events or social activities), and individually numbered to permit analysis by site. Ambassadors were given inexpensive pens to provide to respondents as giveaways in exchange for their participation. Program staff also provided onsite technical assistance to ambassadors on request in their respective houses of worship.

Ambassadors used convenience sampling methods to obtain assessments from any member of their congregation. Congregation members used worship services and other events (eg, health fairs, meal programs, Bible study, affinity groups) to obtain completed instruments. The final response rate was 97%; houses of worship submitted most of their assessment cards by April 2012. Data were analyzed by using SPSS version 19.0 (IBM Corporation, Armonk, New York). Each house of worship received a data report (Appendix B), which was used to provide a snapshot of the house of worship’s climate at baseline and to assist ambassadors in the identification of areas of focus during program implementation.

## Interpretation

Recent research has highlighted various community and institutional factors that can determine the success or failure of program implementation (17,18). Providing a well-funded initiative allowed program staff to build long-term relationships. Through funding, 1 staff member was involved with all aspects of the program, including recruitment, implementation, and evaluation through all 3 phases. Consistent staff (from the period of funding receipt through the recruitment and congregation assessment periods) allowed relationships to evolve and deepen, as program staff learned the nuances of the various houses of worship, and ambassadors had a clearly identified, accessible staff member to reach if needed.

The use of informal open houses and a formal orientation session allowed program staff to reach lay members in person and explain the components of the program and how it complements the existing mission(s) of their faith-based organizations. Elements of program phases were designed to help build ambassadors’ skills and their self-efficacy toward the design of activities that would be successful and well received by their congregations. In-depth training of ambassadors throughout program delivery was helpful to build their data collection and reporting skills, as well as to help them understand why providing data is so important. Ambassadors were given skills and tools through monthly training sessions and individualized technical assistance, but the program was purposely designed not to be prescriptive. This design allowed ambassadors to use their creativity and take ownership of the activities they were conducting within their houses of worship.

This project underscored the importance of community engagement and partnership from the onset, which has been identified as a factor to successful program implementation (17). These factors were instrumental in building relationships with community partners at onset of the program and allowed us to identify and fast track our network building with like-minded partners.

Similar to the experience of other health education programs delivered in community settings, a limitation of our program was the inability to reach as many houses of worship as we wanted. Even with a large mailing, informal open houses, and follow-up contacts from trusted community partners, we attracted a less-than-ideal sample of houses of worship to apply for participation in the program. We learned through our recruitment process that some houses of worship were experiencing various issues, such as dwindling memberships or worship service attendance, reduced revenue, growing urgent needs from their congregations (eg, social programs, financial hardships), and changes in or absence of leadership during the recruitment or promotion period. In some cases, our introductory letters may have been misplaced or discarded. Some of these issues have been highlighted as barriers in other implementation research, and we recognize that participating in our program may not have been a priority for houses of worship with larger institutional concerns (13).

Through implementing this program, we learned lessons and would make some changes if it were to be replicated. This program was implemented with only 1 full-time staff member, supported by a host of community partners and volunteers. Additional staff would have allowed more in-person visits to houses of worship during the recruitment phase. Research has shown that such visits may increase program interest among clergy leadership, because people often prefer face-to-face interaction, especially when encountering organizations with which they are not familiar. Although we were fortunate to have a consistent staff member, additional staff may have increased our reach and thus increased the number of houses of worship who applied for participation (5).

In future implementation, we would provide incentives to both the houses of worship and ambassadors for their commitment. Research suggests that financial incentives can be helpful in providing additional support and keeping organizations engaged (13,19,20). Our adapted program was designed to provide a financial incentive to a house of worship for successful completion of each phase, but this incentive may not be enough to interest some ambassadors in providing their time for participation. Providing support at the individual and institutional levels may help houses of worship consider applying for participation in the program.

Despite these limitations, our results contribute to the body of knowledge regarding the use of faith-based organizations to provide health education. This case study highlights the successes and challenges of recruiting volunteer lay members for program implementation and helps identify elements that should be incorporated to maximize participant recruitment in future programming.

## Figures and Tables

**Figure:**
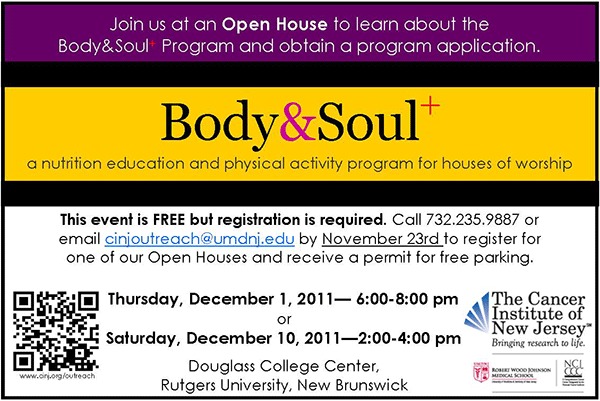
This is a sample of a promotional card for two Body and Soul-plus events held in December 2011 by Rutgers Cancer Institute of New Jersey (formerly known as The Cancer Institute of New Jersey).

There is also a QR code that a reader can scan to obtain this information from a website.

## Appendix B. Body and Soul Sample Congregation Assessment Report

This report is available for download as a Microsoft Word document.

## References

[1] US Department of Agriculture, US Department of Health and Human Services. Dietary Guidelines for Americans, 2010. Washington (DC): US Government Printing Office; 2010.

[2] Cancer prevention and early detection facts and figures 2013. Atlanta (GA): American Cancer Society; 2013.

[3] Policy and action for cancer prevention: food, nutrition, and physical activity: a global perspective. Washington (DC): World Cancer Research Fund, American Institute for Cancer Research; 2009.

[4] Kushi LH , Doyle C , McCullough M , Rock CL , Demark-Wahnefried W , Bandera EV , American Cancer Society Guidelines on nutrition and physical activity for cancer prevention: reducing the risk of cancer with healthy food choices and physical activity. CA Cancer J Clin 2012;62(1):30–67. 10.3322/caac.20140 22237782

[5] Campbell MK , Hudson MA , Resnicow K , Blakeney N , Paxton A , Baskin M . Church-based health interventions: evidence and lessons learned. Annu Rev Public Health 2007;28:213–34. 10.1146/annurev.publhealth.28.021406.144016 17155879

[6] Behavioral Risk Factor Surveillance System public use data tape 2011. Atlanta (GA): Centers for Disease Control and Prevention, National Center for Chronic Disease Prevention and Health Promotion; 2012.

[7] County health rankings and roadmaps 2012: a healthier nation, county by county. University of Wisconsin Population Health Institute. http://www.countyhealthrankings.org. Accessed July 4, 2013.

[8] Campbell MK , Demark-Wahnefried W , Symons M , Kalsbeek WD , Dodds J , Cowan A , Fruit and vegetable consumption and prevention of cancer: the Black Churches United for Better Health project. Am J Public Health 1999;89(9):1390–6. 10.2105/AJPH.89.9.1390 10474558 PMC1508774

[9] Campbell MK , Motsinger BM , Ingram A , Jewell D , Makarushka C , Beatty B , The North Carolina Black Churches United for Better Health Project: intervention and process evaluation. Health Educ Behav 2000;27(2):241–53. 10.1177/109019810002700210 10768805

[10] Resnicow K , Jackson A , Wang T , De AK , McCarty F , Dudley WN , A motivational interviewing intervention to increase fruit and vegetable intake through black churches: results of the Eat for Life trial. Am J Public Health 2001;91(10):1686–93. 10.2105/AJPH.91.10.1686 11574336 PMC1446855

[11] Resnicow K , Wallace DC , Jackson A , Digirolamo A , Odom E , Wang T , Dietary change through African American churches: baseline results and program description of the Eat for Life trial. J Cancer Educ 2000;15(3):156–63. 11019764 10.1080/08858190009528685

[12] Resnicow K , Jackson A , Blissett D , Wang T , McCarty F , Rahotep S , Results of the healthy body healthy spirit trial. Health Psychol 205;24(4):339-48. 10.1037/0278-6133.24.4.339 16045368

[13] Baruth M , Wilcox S , Laken M , Bopp M , Saunders R . Implementation of a faith-based physical activity intervention: insights from church health directors. J Community Health 2008;33(5):304–12. 10.1007/s10900-008-9098-4 18473154

[14] Bopp M , Wilcox S , Laken M , Hooker SP , Saunders R , Parra-Medina D , Using the RE-AIM framework to evaluate a physical activity intervention in churches. Prev Chronic Dis 2007;4(4):A87. 17875262 PMC2099285

[15] Research to Reality. National Cancer Institute, US National Institutes of Health. https://researchtoreality.cancer.gov/. Accessed July 3, 2013.

[16] Health Information National Trends Survey 3. Washington (DC): National Cancer Institute, National Institutes of Health; 2007.

[17] Allicock M , Campbell MK , Valle CG , Carr C , Resnicow K , Gizlice Z . Evaluating the dissemination of Body & Soul, an evidence-based fruit and vegetable intake intervention: challenges for dissemination and implementation research. J Nutr Educ Behav 2012;44(6):530–8. 10.1016/j.jneb.2011.09.002 22406012 PMC3374882

[18] Durlak JA , DuPre EP . Implementation matters: a review of research on the influence of implementation on program outcomes and the factors affecting implementation. Am J Community Psychol 2008;41(3-4):327–50. 10.1007/s10464-008-9165-0 18322790

[19] Demark-Wahnefried W , McClelland JW , Jackson B , Campbell MK , Cowan A , Hoben K , Partnering with African American churches to achieve better health: lessons learned during the Black Churches United for Better Health 5 a day project. J Cancer Educ 2000;15(3):164–7. 11019765 10.1080/08858190009528686

[20] Baskin ML , Resnicow K , Campbell MK . Conducting health interventions in black churches: a model for building effective partnerships. Ethn Dis 2001;11(4):823–33. 11763307

