# Supplementary figures and images for: Engaging the Community to Improve Nutrition and Physical Activity Among Houses of Worship
Source: Prev Chronic Dis. 2014 Mar 13;11:E38. doi: 10.5888/pcd11.130270 (PMC3958142; doi:10.5888/pcd11.130270)

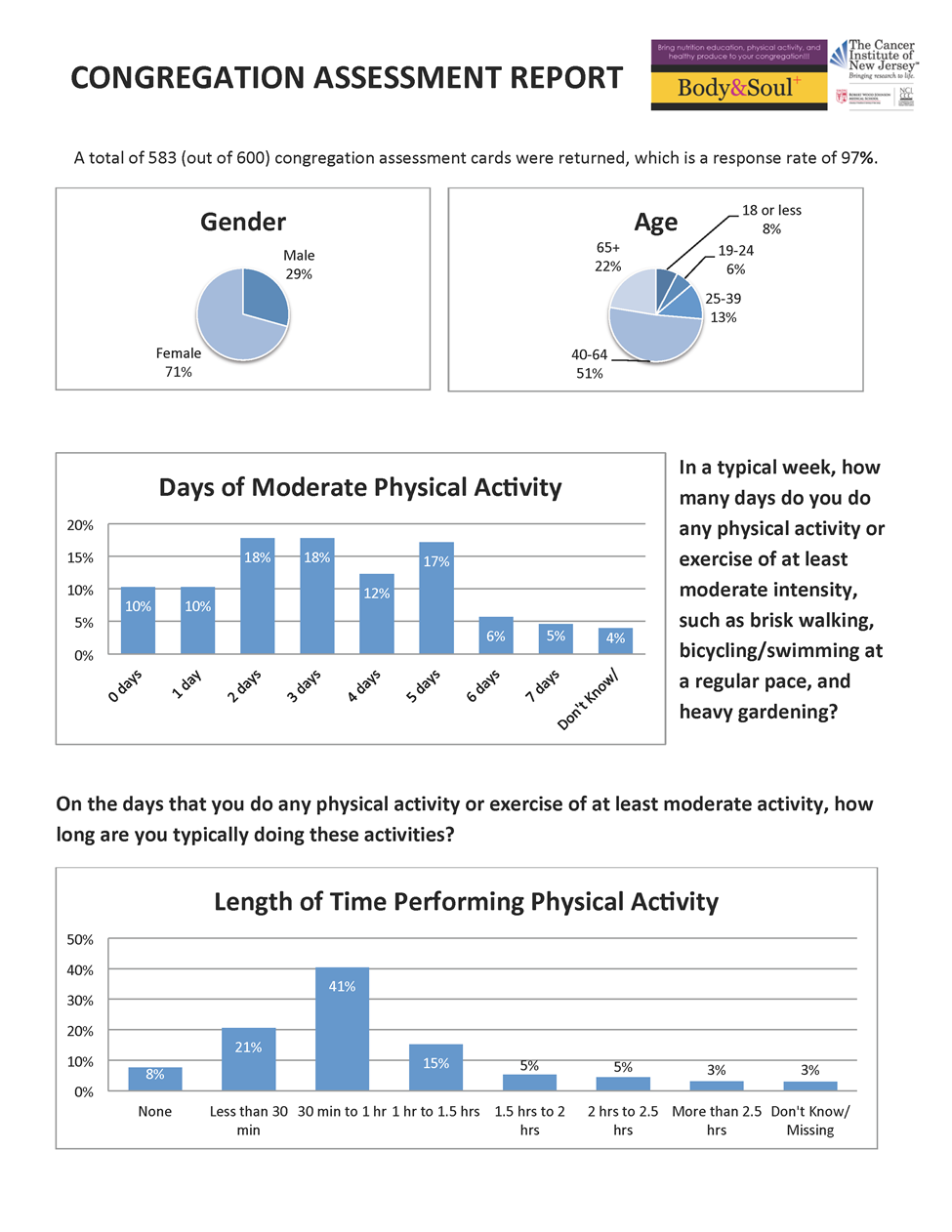


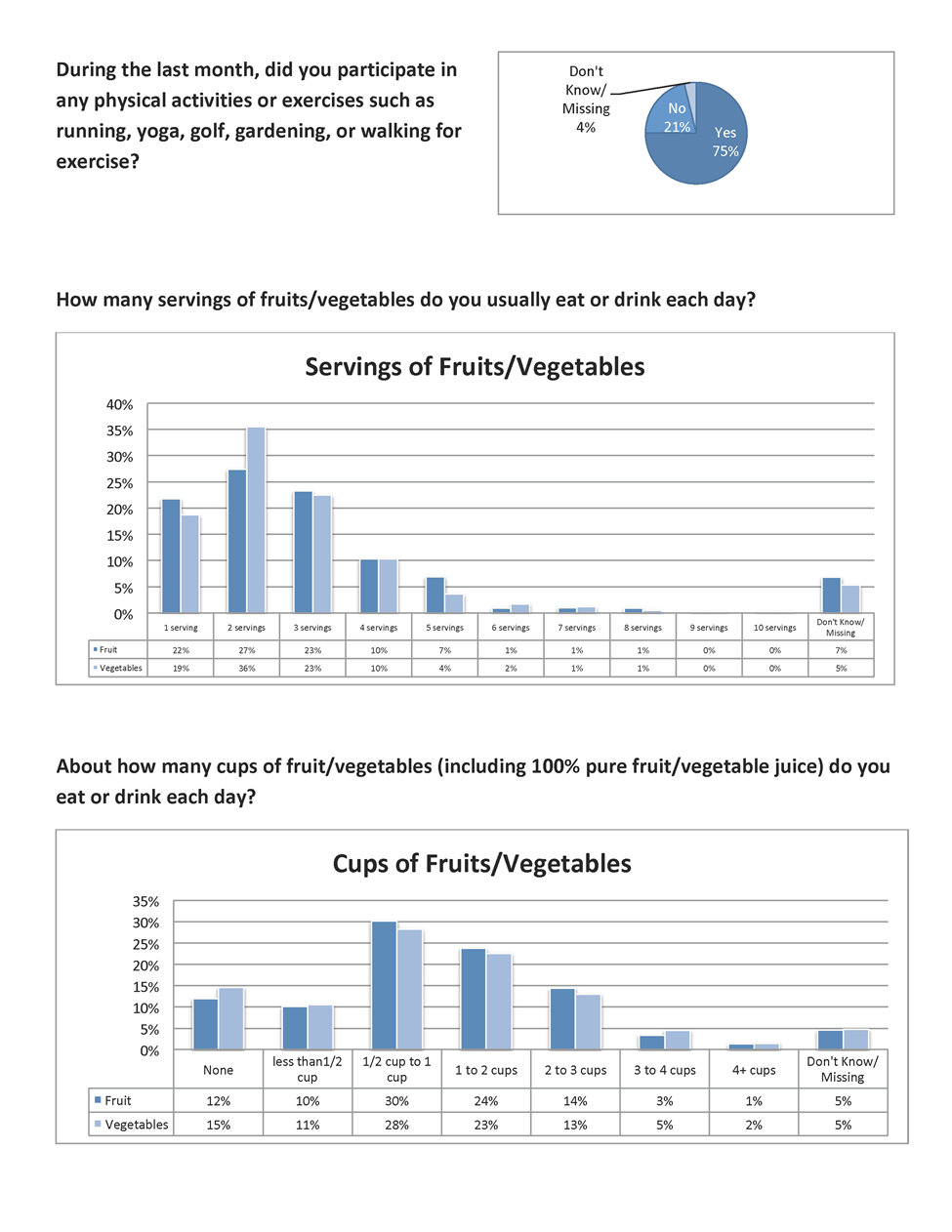

Supplement: Supplementary file 1 [file 13_0270_01.doc]
